# Low frequency conduction block: a promising new technique to advance bioelectronic medicines

**DOI:** 10.1186/s42234-021-00073-9

**Published:** 2021-07-26

**Authors:** Silvia V. Conde

**Affiliations:** 1 iNOVA4Health, CEDOC, NOVA Medical School, NMS, Faculdade Ciências Médicas, Universidade Nova de Lisboa, 1169-056 Lisboa, Portugal; 2 CEDOC, Centro Estudos Doenças Crónicas, NOVA Medical School, Faculdade de Ciências Médicas, Universidade Nova de Lisboa, Rua Câmara Pestana, nº6, 6A, Edifício CEDOC II, piso 3, 1150-082 Lisboa, Portugal

**Keywords:** low frequency alternating current, reversibility, efficacy, onset response, high frequency stimulation

## Abstract

Nerve conduction block is an appealing way to selective target the nervous system for treating pathological conditions. Several modalities were described in the past, with the kilohertz frequency stimulation generating an enormous interest and tested successfully in clinical settings. Some shortcomings associated with different modalities of nerve blocking can limit its clinical use, as the “onset response”, the high demand of energy supply, among others. A recent study by Muzquiz and colleagues describes the efficacy and reversibility of low frequency alternating currents in blocking the cervical vagus in the pig, in the absence of an onset effect and apparent lack of neuronal damage.

Electrical stimulation of nerves has been used for years, experimentally and clinically, to modulate excitability of cells and neurocircuitries within the body. One of the modalities of electrical stimulation, for which there has been an enormous interest, is the high frequency stimulation, which uses electrical frequencies within the range of 1–50 kHz to achieve reversible nerve conduction block(Patel et al. 2018). Seeking its clinical potential within the bioelectronic medicines field, kilohertz frequency stimulation to induce nerve conduction block was tested with success, in several peripheral nerves to tackle pathological conditions as obesity(Apovian et al. 2017; Sarr et al. 2012)and type 2 diabetes(Sacramento et al. 2018), to improve bladder function(Boger et al. 2008; Boger et al. 2012)and for modulation of chronic pain(Taylor et al. 2020; Youn et al. 2015). High frequency stimulation have several characteristics that makes it extremely attractive to be used as a nerve blocker as: (1) its reversibility in milliseconds(Sacramento et al. 2018; Kilgore et al. 2004; Fjordbakk et al. 2019); (2) its efficacy in large diameter nerves in large species (for a review see(Patel et al. 2018)) and in long-term(Sacramento et al. 2018); (3) the absence of neuronal damage(Sacramento et al. 2018; Ling et al. 2019)and (4) the possibility of providing partial nerve block(Patel et al. 2018; Bhadra et al. 2005). However, it exhibits some shortcomings that may hinder its clinical use. In this regard, kilohertz frequency stimulation has been associated to a transient hyperactivity of the nerve named “onset response”(Bhadra et al. 2005)that may bring unwanted physiological effects. To overcome onset responses-associated with kilohertz high frequency stimulation several actions have been attempted, as the alteration in current waveforms and electrode geometry or even the combination of different electrodes and types of currents(Patel et al. 2018; Peña et al. 2020). For instance, Sacramento et al.(Sacramento et al. 2018) described that 50 kHz, 2 mA applied via cuff electrodes bilaterally in rectangular pulses to the carotid sinus nerve, in rats, did not evoke a transient increase in the activity of this nerve, an observation of clinical importance for bioelectronics modulation to treat metabolic diseases.

Another shortcoming of kilohertz high frequency stimulation usage and its applicability to the clinics is the energy demand and the hardware needed to supply continuous kilohertz frequencies and high amplitudes to achieve nerve conduction block, particularly when chronic continuous nerve blocking is required to reverse a pathological condition.

Recently, a new modality to achieve nerve conduction block with the potential to mitigate some of the shortcomings related with high frequency blocking and therefore with high potential for clinical use has been described (Muzquiz et al. 2021). Low frequency alternating current waveform (1 Hz) at the cervical vagus, reversibly blocks in 80 % the bradycardic activity elicited by vagal stimulation in rats, without the presence of onset response and with no apparent injury to the nerve (Mintch et al. 2019). But most importantly, in this edition of “Bioelectronic Medicine” Muzquiz et al.^16^ (2021) demonstrates the efficacy and reversibility of low frequency conduction block on larger caliber myelinated vagal afferent fibers in the swine. The authors tested 1 Hz sinusoidal current waveform delivered through a bipolar nerve cuff electrode, placed unilaterally to the left cervical vagus nerve, on the ability of nerve to evoke compound action potentials and to produce changes in breathing rate mediated by the Hering-Breuer reflex in anaesthetized swines. They found that cervical vagus nerve low frequency block at current levels of 1.1 ± 0.3 mAp (current to peak), which were within the water window of the working electrode, were able to decrease in 87 % the vagal stimulated reduction in breathing rate through the Hering-Breuer reflex, effects immediately reversed upon unblocking of the nerve, and without any onset response and nerve damage^16^ (Muzquiz et al. 2021). Moreover, they found while monitoring vagus nerve activity that cervical vagus low frequency blocking slow down and reduced the amplitude of components of the compound nerve action potentials, being these changes correlated with the effectiveness of low frequency blocking. Reversible nerve blockade with low frequency alternate current rises as an appealing alternative to high frequency block due to its rapid reversibility, the absence of onset response and its low-threshold characteristics. Nevertheless, further studies are needed to completely elucidate the mechanisms behind nerve block with low frequency alternative current block and to elucidate if this modality might has differential effects on myelinated and unmyelinated fibers. Moreover, of particular importance is understanding if low frequency alternating current block is effective in producing nerve block in other nerves apart from the vagus and in other large species as well as to prove its efficacy and reversibility for long-term use. These clarifications will allow moving low frequency alternative currents usage to promote nerve conduction block closer to the clinics.

## Data Availability

Non-applicable.
